# Identification of Key Metabolites in Poly-γ-Glutamic Acid Production by Tuning γ-PGA Synthetase Expression

**DOI:** 10.3389/fbioe.2020.00038

**Published:** 2020-01-30

**Authors:** Birthe Halmschlag, Sastia P. Putri, Eiichiro Fukusaki, Lars M. Blank

**Affiliations:** ^1^Institute of Applied Microbiology-iAMB, Aachen Biology and Biotechnology-ABBt, RWTH Aachen University, Aachen, Germany; ^2^Department of Biotechnology, Graduate School of Engineering, Osaka University, Osaka, Japan

**Keywords:** biopolymer, *Bacillus subtilis*, natto, metabolomics, natural product

## Abstract

Poly-γ-glutamic acid (γ-PGA) production is commonly achieved using glycerol, citrate, and L-glutamic acid as substrates. The constitutive expression of the γ-PGA synthetase enabled γ-PGA production with *Bacillus subtilis* from glucose only. The precursors for γ-PGA synthesis, D- and L-glutamate, are ubiquitous metabolites. Hence, the metabolic flux toward γ-PGA directly depends on the concentration and activity of the synthetase and thereby on its expression. To identify pathway bottlenecks and important metabolites that are highly correlated with γ-PGA production from glucose, we engineered *B. subtilis* strains with varying γ-PGA synthesis rates. To alter the rate of γ-PGA synthesis, the expression level was controlled by two approaches: (1) Using promoter variants from the constitutive promoter P_*veg*_ and (2) Varying induction strength of the xylose inducible promoter P_*xyl*_. The variation in the metabolism caused by γ-PGA production was investigated using metabolome analysis. The xylose-induction strategy revealed that the γ-PGA production rate increased the total fluxes through metabolism indicating a driven by demand adaption of the metabolism. Metabolic bottlenecks during γ-PGA from glucose were identified by generation of a model that correlates γ-PGA production rate with intracellular metabolite levels. The generated model indicates the correlation of certain metabolites such as phosphoenolpyruvate with γ-PGA production. The identified metabolites are targets for strain improvement to achieve high level γ-PGA production from glucose.

## Introduction

Poly-γ-glutamic acid (γ-PGA) is an anionic, biodegradable, non-toxic polymer composed of D- and L-glutamic acid units linked by γ-glutamyl bonds. γ-PGA production with *Bacillus subtilis* is mainly known from *B. subtilis* (natto) in the production of natto, a traditional Japanese dish. But *B. subtilis* has also been considered as a promising organism for industrial production of γ-PGA. In case of glutamate-independent production of γ-PGA, *B. subtilis* C1 has been used with glucose and citrate as substrates resulting in a maximal titer of 21.4 g L^–1^ γ-PGA (Shih et al., 2005). Higher γ-PGA titers have been achieved by process optimization. The use of several substrates for γ-PGA production and different process strategies like fed-batch cultivations have been reported (Ogawa et al., 1997; Yoon et al., 2000). Mostly wild-type γ-PGA producers like *B. subtilis* (natto) (Ogawa et al., 1997) and *Bacillus licheniformis* ATCC9945 (Cromwick et al., 1996) have been used for the production focusing rather on process optimization than on metabolic engineering strategies to achieve a more efficient production. In order to find metabolic engineering targets to enhance γ-PGA production, a genetically easily amenable host organism is favorable.

The synthesis of γ-PGA is catalyzed by an enzyme complex, the γ-PGA synthetase, which is encoded by the *pgs* operon. The operon consists of four genes: *pgsB, pgsC, pgsA*, and *pgsE.* Moreover, one hydrolyzing enzyme, the γ-PGA depolymerase is encoded by a gene located directly downstream of the *pgs* genes. The expression of *pgs* genes is both sufficient and necessary to achieve γ-PGA production in *B. subtilis* (Urushibata et al., 2002). The expression in *B. subtilis* 168 can be achieved by either deleting the mutations in the *degU* and the *swrA* gene encoding for the expression regulator DegU and the swarming motility factor, respectively (Ohsawa et al., 2009), or by controlling the expression from an alternative promoter (Urushibata et al., 2002).

During γ-PGA production, glutamate is a central metabolite. Glutamate is used as cell wall constituent in its D-enantiomeric form, as amino acid for protein synthesis in its L-form and finally, for γ-PGA production in both forms. Taking into account that glutamate also plays an important role in linking carbon and nitrogen metabolism, its production and turnover has to be carefully regulated. Hence, the overproduction of γ-PGA can be seen as metabolic burden for the cells. Therefore, it is important to clarify the influence of the γ-PGA synthetase amount, which can be controlled by the promoter strength, as well as the influence of γ-PGA overproduction on the metabolism. In many cases the gene expression is only evaluated at the points of knockout or overexpression, but the whole range of effects can only be explained by specifically tuning the gene expression (Alper et al., 2005). To fine tune gene expression, promoter libraries for pro- and eukaryotic promoters have been created (Jensen and Hammer, 1998; Blazeck and Alper, 2013; Redden et al., 2015). The promoter located upstream of the vegetative genes (P_*veg*_) is known as a strong promoter of *B. subtilis*. Based on this promoter, a promoter library harboring changes in the −10 region of the promoter has been created (Guiziou et al., 2016). For these promoter variants, the activity of the promoter was even increased. In the case of γ-PGA production, it might also be of interest to use weaker promoters in order not to increase the demand on glutamate too excessively. For the development of a promoter library, the exact activity of a promoter variant can be tested based on the expression of a reporter gene. The use of the Green Fluorescing Protein (GFP) as reporter allows for the selection of promoter variants based on the measured fluorescence (Chalfie et al., 1994). The *gfpmut3* gene encodes a stable variant of GFP (Cormack et al., 1996). Besides promoter libraries, the expression of specific genes may also be controlled by the use of an inducible promoter. The *B. subtilis* xylose promoter (P_*xyl*_) is transcriptionally repressed by XylR and induced by xylose (Gärtner et al., 1988). Hence, the strength of gene expression can be controlled by P_*xyl*_ and varying inducer concentrations.

Recent advances in the design of whole-cell biocatalysts focus on rational approaches (Kuhn et al., 2010). The integration of omics data strongly supports the biocatalyst development (Joyce and Palsson, 2006; Tyo et al., 2007). Especially the field of metabolomics is widely used to guide metabolic engineering efforts (Putri et al., 2013). The fine-tuning of gene expression by promoter strength enables the control of metabolic flux on the most fundamental level. Metabolome analysis of strains with varying flux can be used to identify key metabolites in production strains. The identified metabolites offer targets for metabolic engineering in order to cure metabolic bottlenecks of producer strains and thereby increase the production by targeted strain design.

In this study, we investigated the metabolic response of *B. subtilis* in dependence of *pgs* expression. The laboratory strain *B. subtilis* 168 has been reported as favorable γ-PGA production host due to its natural competence and well-investigated genetics and physiology (Halmschlag et al., 2019). Two alternative promoters were used, the constitutive P_*veg*_ and variants thereof and the inducible P_*xyl*_ promoter. In depth metabolome analysis revealed strong responses of metabolism to γ-PGA production, while homeostasis for glutamate. The results can guide targeted strain design efforts for the intensified production of γ-PGA.

## Materials and Methods

### Reagents

D-Glucose, L-glutamic acid, NH_4_Cl, K_2_HPO_4_, MgSO_4_ × 7H_2_O, CaCl_2_ × 2H_2_O, MnSO_4_ × H_2_O, FeCl_3_ × 6H_2_O, ZnSO_4_ × 7H_2_O, Na_2_-EDTA, CuSO_4_ × 5H_2_O and CoCl_2_ × 6H_2_O were purchased from Carl Roth GmbH + Co., KG (Karlsruhe, Germany). LC/MS-grade ultra-pure water, HPLC-grade chloroform, acetic acid, H_2_SO_4_ and NH_4_HCO_3_ were purchased from Wako Pure Chemical Industries, Ltd. (Osaka, Japan). 10-Camphorsulfonic acid and tributylamine were purchased from SigmaAldrich (St. Louis, MO, United States).

### Strains, Plasmids and Growth Conditions

The bacterial strains and plasmids used and created in this study are listed in Table 1. All cloning steps were carried out in Escherichia *coli* DH5α. The recombinase positive *E. coli* strain JM101 was used to obtain plasmids for the transformation of *B. subtilis*. The strain *B. subtilis* Δspo (Halmschlag et al., 2019) was used for the development of the promoter library and as production host for γ-PGA production.

**TABLE 1:** Strains and plasmids used in this study.

Strain	Genotype/properties	Reference/source
**Strains**		
*E. coli* DH5α	*fhuA*2 Δ (*argF-lacZ*)U169 *phoA glnV*44 Φ80 Δ(*lacZ*)M15 *gyrA*96 *recA*1 *relA*1 *endA*1 thi-1 hsdR17	Meselson and Yuan, 1968
*E. coli* JM101	*glnV*44 thi-1 Δ(*lac-proAB*) F′ [*lacI^*q*^Z*Δ*M15 traD*36 *proAB*^+^]	Messing et al., 1981
*B. subtilis* IIG-Bs2	ΔSPβΔ*skin* ΔPBSX ΔproΦ1 Δ*pks*:CmR, ΔproΦ3 *trp* + Δ*manPA*:*erm* Δ*bpr* Δ*sigG* Δ*sigE* Δ*spoGA*	Wenzel and Altenbuchner, 2015
*B. subtilis*Δspo	Δ*bpr* Δ*sigG* Δ*sigE* Δ*spoGA*	Halmschlag et al., 2019
*B. subtilis* ΔxylAB	Δ*bpr* Δ*sigG* Δ*sigE* Δ*spoGA*Δ*xylAB*	This study
*B. subtilis* PG1	P_*veg*_-pgs	This study
*B. subtilis* PG16 (PV35.1)	Δ*bpr* Δ*sigG* Δ*sigE* Δ*spoGA* PV35.1-pgs	Halmschlag et al., 2020
*B. subtilis* (PV35.3)	Δ*bpr* Δ*sigG* Δ*sigE* Δ*spoGA* PV35.3-pgs	This study
*B. subtilis* (PV35.26)	Δ*bpr* Δ*sigG* Δ*sigE* Δ*spoGA* PV35.26-pgs	This study
*B. subtilis* PG10	Δ*xylAB* Δ*bpr* Δ*sigG* Δ*sigE* Δ*spoGA* Pxyl-pgs	Halmschlag et al., 2019
**Plasmids**		
pJOE-8739	Backbone for markerless counterselection system	Wenzel and Altenbuchner, 2015
pBS-20	Backbone for promoter library integration into *amyE* locus	This study
pBS-21	Template for promoter library construction, *amyE*_up-P_*veg*_-*gfpmut3*-*amyE*_down	This study
pBS-21-PV35.01	PV35.01-*gfpmut3* promoter integration into *amyE* locus	This study
pBS-21-PV35.03	PV35.03-*gfpmut3* promoter integration into *amyE* locus	This study
pBS-21-PV35.01	PV35.26-*gfpmut3* promoter integration into *amyE* locus	This study
pRJ-8-PV35.01	PV35.01 promoter integration upstream of *pgs*	This study
pRJ-8-PV35.03	PV35.03 promoter integration upstream of *pgs*	This study
pRJ-8-PV35.26	PV35.26 promoter integration upstream of *pgs*	This study

For plasmid construction and counter selection, all strains were cultivated at 37°C in LB medium containing 100 μg/mL spectinomycin or 0.5% (w/v) mannose when required. For γ-PGA production and metabolome analysis, the *B. subtilis* strains were grown in glucose minimal medium.

### *Pgs* Expression Induction With Xylose

For the use of xylose as inducing agent, the *xylAB* genes encoding the xylose isomerase and xylulokinase were deleted in the background of strain *B. subtilis* Δspo. The thereby obtained strain is not capable of metabolizing xylose. Subsequently, the native P_*xyl*_ promoter was integrated into the genome of *B. subtilis* Δspo Δ*xylAB* upstream of the *pgs* operon. For that purpose the plasmid pBs-02 was generated. The plasmid contains the backbone of pJOE8739 linearized with primers BS-25/26 (Supplementary Table S1), P_*xyl*_ amplified with BS-09/10 and two integration sites up- and downstream of the native promoter P_*pgs*_ that were amplified with the primer pairs TS1_fwd/TS1_rev and TS2_fwd/TS2_rev. The created strain expressing *pgs* under control of the xylose-inducible promoter Pxyl was designated as *B. subtilis* PG10.

### Promoter Library Construction

The reporter gene *gfpmut3* was amplified from plasmid pBSMul1-gfpmut3 with primer pair BS-111/BS-112. A part of the *amyE* locus was amplified with BS-113/-114 to be used as homologous sequence for integration. For the assembly of plasmid pBS-21, the vector backbone pBs-20 was linearized with BS-25/-26. The backbone includes a spectinomycin resistance gene (*SpcR*), an origin of replication for *E. coli*, the *rop* gene with regulator function for replication and the second part of the *amyE* locus for homologous recombination into the *B. subtilis* genome. The native promoter P_*veg*_ was amplified from genomic *B. subtilis* DNA using the primers BS-109 and BS-110. The promoter fragment was fused with the *gfpmut3* gene, the *amyE* upstream, and the pBs-20 based backbone by DNA HiFi Assembly (NEB, Frankfurt am Main, Germany). The thereby constructed plasmid pBs-21 was used as template to amplify two fragments, one containing the first homologous part (*amyE*-front) and the spectinomycin resistance gene (*SpcR*) and the other one containing the promoter, *gfp* gene and the second homologous part (*amyE*-back). The two fragments were amplified using the primer pairs BS-115/-116 and BS-117b/118, respectively. The primer BS-117b was a degenerated primer containing the NNN sequence to vary the −35 box sequence of the promoter. After fusion of the fragments by PCR and cloning into the pJET vector, *Bacillus* was transformed with the plasmids including promoter variants.

### Determination of Promoter Activity

The *B. subtilis* colonies with varying promoter sequences were analyzed with respect to the promoter activity using the BioLector (m2p-labs, Baesweiler, Germany). The biomass was measured at 620 nm extinction/emission at gain 50. The fluorescence of GFP was detected with 488 nm extinction and 520 nm emission at gain 70. All colonies were analyzed in triplicates and the promoter activity was calculated as mean of the slope calculated as quotient of increase in fluorescence and increase in biomass during the exponential phase.

### Integration of Promoter Variants for *pgs* Operon

The promoter sequence information was obtained by sequencing (Eurofins, Ebersberg, Germany). The observed sequences were introduced into pRJ-8 variants by amplification of the complete plasmid using specific primers (BS-180, BS-193, and BS-182 for variant PV35.1, PV35.3, and PV35.26, respectively) with mismatches in the promoter region. The thereby created linear plasmids were phosphorylated with T4 polynucleotide kinase (NEB, Frankfurt am Main, Germany) for 30 min at 37°C and subsequently ligated with T4 ligase (NEB, Frankfurt am Main, Germany). First, *E. coli* DH5α was transformed with these plasmids. The plasmids were purified and the promoter sequences were determined. Second, *B. subtilis* was transformed with those three plasmids. Successfully transformed colonies were selected on LB agar plates containing spectinomycin. The correct integration was checked by PCR and sequencing.

### Cultivation for γ-PGA and Metabolite Analysis

For the cultivation of *B. subtilis* glucose was used as sole carbon source in a minimal salt medium for the main cultures. The minimal medium contained per liter: 20 g glucose, 7 g NH_4_Cl, 0.5 g MgSO_4_, 0.15 g CaCl_2_, 0.1 g MnSO_4_, 0.04 g FeCl_3_, and 1 mL of a trace element solution. The trace element solution [modified trace element solution after: (Wenzel et al., 2011)] contained per liter: 0.54 g ZnSO_4_^∗^7 H_2_O, 30.15 g Na_2_-EDTA, 0.48 g CuSO_4_^∗^5 H_2_O and 0.54 g CoCl_2_^∗^6 H_2_O. The medium was buffered using 0.1 M potassium phosphate buffer at pH 7. All media components were sterilized by autoclaving for 20 min at 121°C except for the trace element solution, which was sterile filtered. For xylose induction of *pgs* expression with *B. subtilis* PG10, xylose was added in concentrations of 0, 1, 5, 10, 15, 25, 40, 50, 80, 100, and 200 mM. The first pre-culture in LB medium was inoculated from a glycerol stock. The LB medium contained 10 g L^–1^ tryptone, 5 g L^–1^ yeast extract, and 10 g L^–1^ NaCl at pH 7.4. A second pre-culture in glucose minimal medium was inoculated with cells from the LB medium and grown overnight. The main cultures were inoculated to an OD_600_ of 0.05 from pre-culture. The cultivations were carried out in 250 mL Erlenmeyer flasks filled with 25 mL medium. The cultures were incubated on a rotary shaker (Bio-Shaker BR-3000LF, Taitec, Saitama, Japan) at 37°C, 200 rpm, 25 mm shaking diameter. All cultivations were performed in triplicates whereas the reported data represents the mean. The cell growth was monitored by OD_600_ measurements of samples taken from the shake flasks using the GeneQuant 100 spectrophotometer (GE Healthcare United Kingdom, Ltd., Buckinghamshire, United Kingdom). Additionally, the cell growth was monitored online with the Cell Growth Quantifier (CGQ; Aquila Biolabs, Baesweiler, Germany). The biomass concentration is given as cell dry weight (CDW) calculated from the OD_600_ using the correlation equation CDW [g L^–1^] = 0.5381^∗^OD_600_ + 0.0074.

### Analysis of γ-PGA

The production of γ-PGA was analyzed with a cetyltrimethylammonium bromide (CTAB) assay. To purify γ-PGA, the culture broth was centrifuged for 30 min (10,000*g*, 4°C) to separate the cells. The double amount of ethanol was added to 15 mL of supernatant containing γ-PGA to precipitate the γ-PGA (1:2 ratio, sample:ethanol). The solution was incubated at 4°C overnight. By centrifugation for 10 min at 4°C and 10,000*g*, the precipitated γ-PGA was recovered as a pellet. The pellet was resuspended in 1 mL distilled water. The addition of 100 μL 0.07 M CTAB in 2% (w/v) NaOH to 100 μL of γ-PGA sample led to the precipitation of the γ-PGA/CTAB complex resulting in an increased turbidity that was measured in a plate reader (Synergy Mx plate reader, BioTek Instruments, Inc., Winooski, United States) at 400 nm. A calibration curve using a 1 MDa γ-PGA standard [(Henkel AG & Co., KGaA, Düsseldorf, Germany] was used to calculated the γ-PGA concentration in the culture broth. γ-PGA samples were diluted for the CTAB assay to reach the linear range of the assay up to 0.1 g/L γ-PGA (equaling a turbidity of <0.5).

### Sample Preparation for Metabolome Analysis

A sample volume which satisfies the equation sampling volume (mL) ^∗^OD_600_ = 5 was filtered with a PVDF filter (pore size 0.45 mm; Merck Millipore, Burlington, NJ, United States) using vacuum. The filter was washed with the doubled amount of 300 mM NH_4_HCO_3_ (1:2 ratio, sample: NH_4_HCO_3_). Subsequently, the metabolism was quenched by soaking the filter in liquid N_2_. The filter was transferred to a 2 mL sample tube and was stored at −80°C until metabolite extraction. To extract the metabolites, 1.875 mL of extraction solvent (1:2:2 ratio, H_2_O:MeOH:chloroform, including 7 nM 10-camphorsulfonic acid as internal standard) was added to the sample tube including the filter. After vortexing, the sample tube was centrifuged at 16,000 *g* for 3 min at 4°C. 350 μL of the supernatant were transferred to a 1.5 mL sampling tube, concentrated by vacuum centrifugation for 2 h and freeze dried overnight. The pellet was stored at −80°C until analyzed by LC-MS.

### Ion-Pair-LC/MS/MS Analysis

The dried sample is resuspended in 50 μL ultra-pure water and is transferred into a conical glass vial. The Ion-pair-liquid chromatography coupled with tandem mass spectrometry (LC/MS/MS) analysis was performed using a Shimadzu Nexera UHPLC system coupled with an LCMS 8030 Plus device (Shimadzu Co., Kyoto, Japan). The system was equipped with a PE capped CERI L-column 2ODS column (2.1 mm × 150 mm, particle size 3 mm, Chemicals Evaluation and Research Institute, Tokyo, Japan). As mobile phase a gradient of a mixture of solvent A and B is used, where solvent A is 10 mM tributylamine and 15 mM acetate in ultra-pure water and solvent B is pure methanol. The flow rate was set to 0.2 mL min^–1^. For gradient elution of metabolites, starting from 0% concentration of solvent B, the concentration of B was increased to 15% after 1 min with a gradient of 30% min^–1^, hold for 1.5 min, increased to 50% within 5 min and subsequently increased to 100% within 2 min. The 100% solvent B concentration was held for 1.5 min, decreased to 0% from 11.5 min on and held at this concentration for 8.5 min. The column oven temperature was set to 45°C. The MS parameters were as follows: probe position, þ1.5 mm; desolvation line temperature, 250°C; drying gas flow, 15 L/min; heat block temperature, 400°C; and nebulized gas flow, 2 L/min. As a Quality Control (QC) sample, 2 μL of each analyzed sample of a batch were pooled into a vial. For each sample (including the QC sample), 3 μL were injected to the ion-pair-LC/MS/MS for metabolite analysis.

### Data Processing and Analysis

The calculation of the peak area was carried out using MRMPROBS ver. 2.38 and manual inspection of the chromatogram was conducted. The data was normalized according to the peak area of the internal standard, 10-camphorsulfonic acid. SIMCA 13 (Umetrics, Umeå, Sweden) was used for principal component analysis (PCA) and partial least squares projection to latent structures (PLS) analysis.

## Results

To identify metabolites that are highly correlated with γ-PGA production from glucose in *B. subtilis*, intracellular metabolite measurements for strains with varying γ-PGA synthesis rates were carried out. The variation in γ-PGA synthesis rate was achieved by two approaches. First, the expression of the γ-PGA synthetase was controlled by constitutive promoters of different strengths. The promoter strength was determined by fluorescence measurements using *gfp* as reporter gene. Second, the inducible P_*xyl*_ promoter naturally controlling the *xylAB* operon of *B. subtilis* for xylose utilization was employed. The increase in expression of the xylose promoter correlates positively with the xylose concentration in the medium.

### Integration of Promoter Variants for *pgs* Genes and γ-PGA Production

For the promoter library approach, three newly identified promoter variants based on the P_*veg*_ promoter were successfully integrated upstream of the *pgs* genes to control the expression of γ-PGA synthetase and subsequently to vary the γ-PGA production rate. The variants were constructed by changing the -35 box sequence of the P_*veg*_ promoter (TTGACA). The promoter variants differ from the original P_*veg*_ promoter in one or two positions. For variant PV35.01 (AAGACA), two positions were varied. The large deviation from the consensus sequence resulted in only 10% of the original activity. The change of G to T in the third position decreased the activity by 50 percent in variant PV35.3. Variant PV35.26 (GTGACA) exhibits an increased activity of 120% compared to the original promoter.

**FIGURE 1:**
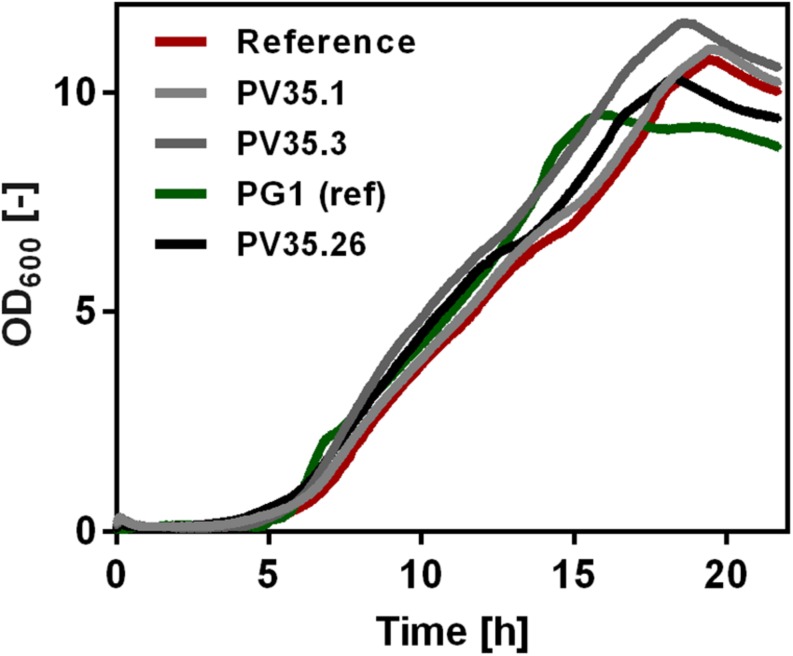
Growth of non-γ-PGA producing *Bacillus subtilis* (reference) and γ-PGA producing *B. subtilis* PV35.1, PV35.3, PG1, and PV35.26. The optical density was determined using the Cell Growth Quantifier (CGQ, Aquila Biolabs, Baesweiler, Germany). The mean of triplicates is shown without standard deviation.

Growth of the created strains was tested by online growth measurements (CGQ) to observe possible influence of higher γ-PGA synthetase expression rates. As shown in Figure 1, the growth curves did not differ significantly. Independent from the promoter activity for *pgs*, all strains grew to comparably high optical densities.

**FIGURE 2:**
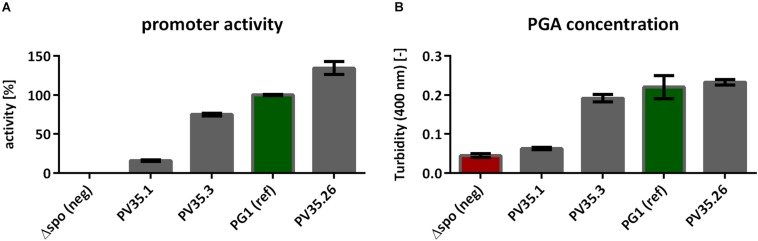
CTAB Assay to determine the γ-PGA concentration based on the measured turbidity. The strains tested were the non-γ-PGA producing negative control *B. subtilis*Δspo and the created strains PV35.1, PV35.3, and PV35.26 with different promoter activities for the *pgs* genes **(A)**. The γ-PGA production was determined during the exponential growth phase (after 9 h, compare Figure 1) using the CTAB assay **(B)**. The promoter activity was calculated from the slope between fluorescence and biomass as determined by the BioLector. The error bars represent the standard deviation.

The titer of γ-PGA production as analyzed by the CTAB assay (Figure 2B) increased with an increasing promoter activity, determined in the BioLector assay (Figure 2A). Hence, the γ-PGA synthesis rate increases with the activity of the promoter controlling the γ-PGA synthetase expression.

### Metabolome Analysis to Reveal Key Metabolites

**FIGURE 3:**
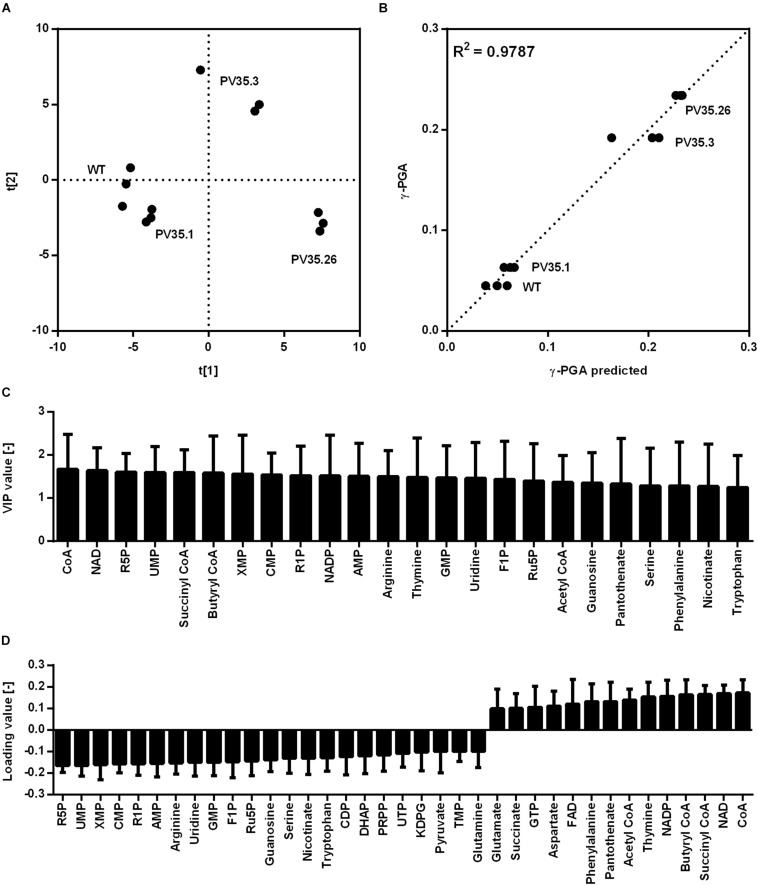
Partial least squares projection to latent structures (PLS) analysis for γ-PGA producing strains based on a promoter library. The analysis was carried out for the non-γ-PGA producing reference (WT) and strains with newly generated promoters PV35.1, PV35.3, and PV35.26 controlling *pgs* expression. Biological triplicates for each strain were cultivated in glucose minimal medium. Samples for analysis were taken during the exponential phase (9 h). The separation according to the metabolom analysis **(A)**, the observed versus predicted γ-PGA values **(B)**,the VIP values **(C)**, and loading values **(D)** for metabolites highly connected to γ-PGA production are shown. The loading data for metabolites with loading values >0.1 are shown whereas negative values correspond to metabolites decreasing with increasing γ-PGA production and metabolites with positive values are increased for higher γ-PGA. ADP, adenosine 5′-diphosphate; AICAR 5′-phosphoribosyl-5-amino-4-imidazolecarboxamide; AMP, adenosine 5′-monophosphate; DHAP, dihydroxyacetone phosphate; G6P, glucose-6-phosphate; KDPG, 2-Keto-3-deoxy-6-phosphogluconate; IMP, Inosine 5′-monophosphate; MEP, methylerythritol 4-phosphate; PRPP, 5-phospho-alpha-D-ribose 1-diphosphate; R1P, ribose-1-phosphate; R5P, ribose-5-phosphate; SBP, sedoheptulose 1,7-bisphosphate; UDP-Glc, uridine 5′-diphosphate-glucose; XMP, xanthosine 5′-phosphate.

The created strains harboring newly generated promoter sequences with different strength were subjected to metabolome analysis to reveal key metabolites that are most correlated with γ-PGA synthesis. For metabolome analysis according to previous studies (Mitsunaga et al., 2016; Fathima et al., 2018), 93 metabolites were detected that are part of the central carbon metabolism including pentose phosphate pathway (PPP), tricarboxylic acid cycle (TCA), and glycolysis intermediates as well as amino acids and nucleotides. The samples were taken after 8 h during exponential growth. The differing γ-PGA concentrations for the tested strains at this time point demonstrate varying γ-PGA synthesis rates. The metabolome data were subjected to partial least square (PLS) analysis (Figure 3). The horizontal separation corresponds to the increasing γ-PGA production from left (low value for PC1) to right (high value for PC1) with R^2^ and Q^2^ of 0.98 and 0.87, respectively. Metabolites that exhibit loading values higher than 0.1, are shown in Figure 3D. Here, negative values correspond to metabolites decreasing with increasing γ-PGA production and metabolites with positive values are increased for higher γ-PGA synthesis rates.

The metabolites with high loading value originate from various metabolic pathways. The most striking loading data for metabolites from the central carbon metabolism is the data for ribose-5-phosphate (R5P), pyruvate, and dihydroxyacetone phosphate (DHAP) with negative values as well as acetyl-CoA with a positive value. Notably, the direct precursors for γ-PGA, glutamate and 2-oxoglutarate hardly change in concentration (loading values of 0.098 and 0.032, respectively). Homeostasis of these metabolites might be explained with their prominent functions in carbon and nitrogen metabolism. While other amino acids such as phenylalanine are increased other amino acids such as serine are lower concentrated during increased γ-PGA production. Serine and R5P are two metabolites which are directly derived from glycolysis intermediates, 3-phosphoglycerate and glucose-6-phosphate (G6P), respectively. The decrease in the metabolite concentrations of serine and R5P can therefore be explained by the higher demand for carbon flux through glycolysis toward the TCA cycle and glutamate. The higher flux toward glutamate is also displayed in the decreased synthesis of precursors for secondary metabolites such as xanthosine 5′-phosphate (XMP). Contrary to the XMP concentration, the acetyl-CoA concentration increases with higher promoter activity. As it was shown for the comparison of a non-γ-PGA producer and a γ-PGA-producer *B. subtilis* PG1, the TCA activity is limiting the precursor supply for γ-PGA production. Meyer et al., demonstrated that citrate synthase, aconitase, and malate dehydrogenase form a protein complex catalyzing sequential reactions of the TCA cycle (Meyer et al., 2011). Additionally, the 2-oxoglutarate dehydrogenase complex and the glutamate synthase are affected by these protein-protein interactions (Meyer et al., 2011). The concentration of succinyl-CoA increases for increasing γ-PGA synthesis rates. With regard to the interaction of TCA cycle enzymes, the higher flux toward 2-oxoglutarate and glutamate likely additionally results in increased succinyl-CoA concentrations. Hence, the metabolome data suggests an increased TCA cycle activity yet being a limiting step for higher γ-PGA production from glucose as sole carbon source. The decreased concentration of R5P for higher promoter activity is surprising with respect to the glucose-6-phosphate dehydrogenase reaction, which is known as main reaction for the supply of NADPH. NADPH is used as cofactor for glutamate synthesis. Therefore, a higher demand for glutamate under γ-PGA producing conditions also results in a higher demand for NADPH. Here, the high positive loading values for both NAD^+^ and NADP^+^ are remarkable, too. Since the applied metabolite extraction method is not suitable for investigating the redox status of the cells, the increasing NAD^+^ concentrations are likely due to a higher demand for NAD(P)H for increasing glutamate *de novo* synthesis.

### Inducible Promoter

**FIGURE 4:**
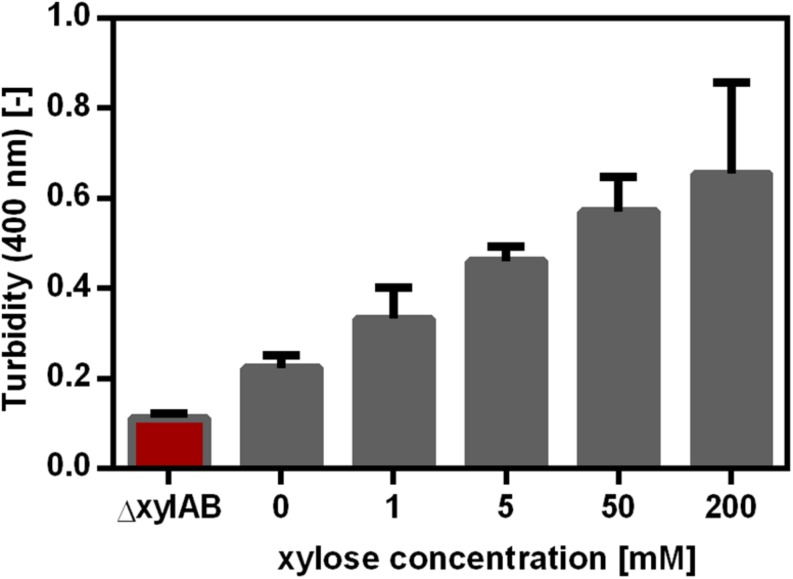
γ-PGA synthesis as a function of xylose concentration for *B. subtilis* PG10. The γ-PGA concentration was determined by the CTAB Assay and is shown as the turbidity at 400 nm. The strains tested were the non-γ-PGA producing negative control *B. subtilis*Δ*xylAB* and *B. subtilis* PG10 with five different xylose concentrations as inducer for *pgs* gene expression. The γ-PGA production was determined during the exponential growth phase (after 9 h, compare Figure 1) for biological triplicates. The error bars represent the standard deviation.

Besides controlling the synthetase expression using a promoter library, the expression was also altered using an inducible promoter. The xylose-inducible promoter P_*xyl*_ controlling the expression of the *xylAB* operon in *B. subtilis* was cloned to control *pgs* expression in *B. subtilis* PG10. The xylose promoter in *B. subtilis* PG10 contained the binding site for the xylose regulator XylR, but not the binding site for catabolite repression via CcpA. These features enabled the induction of the promoter by xylose addition and prevented the repression during growth on glucose. Moreover, the *xylAB* operon was deleted to ensure that xylose is only used as inducer and not as substrate for cell growth. In *B. subtilis*, xylose is imported via the L-arabinose permease encoded by *araE*. The *araE* expression is reported to be arabinose induced and glucose repressed (Sa Nogueira and Ramos, 1997). However, *B. subtilis* Δspo that was used to create the strains in this study grows on xylose without addition of arabinose. Hence, the xylose uptake of the strains used in this study was shown not to depend on arabinose availability. As expected, γ-PGA production increased with increasing xylose concentration (Figure 4). Comparable to the promoter library, a higher synthetase concentration leads to a higher γ-PGA production rate. Controls were the non-γ-PGA producing *B. subtilis* Δspo as well as the *B. subtilis ΔxylAB* used to create PG10, which cannot grow on xylose. In comparison to the promoter library approach, *B. subtilis* PG10 exhibits a lower growth rate (0.43 h^–1^ compared to 0.59 h^–1^). Moreover, the γ-PGA production rate at full induction is higher for the xylose inducible promoter compared to the production rate of the strongest promoter from the library. The determined γ-PGA titers were used to correlate the xylose concentration with γ-PGA production (Figure 4). A logarithmic dependency between the γ-PGA production as represented by the turbidity and the inducer concentration was determined. Since saturation of the production rate was observed for xylose concentrations higher than approximately 100 mM, further concentrations ranging from 0 mM to 50 mM were used for the metabolome analysis to create more variation in the production rate.

**FIGURE 5:**
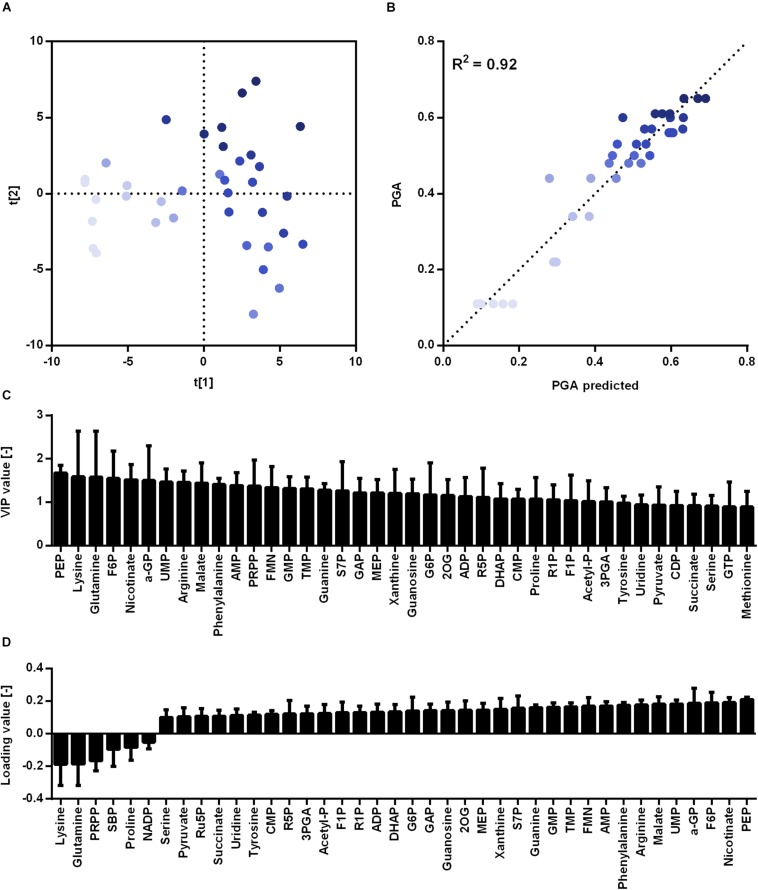
Partial least squares projection to latent structures (PLS) analysis for non-γ-PGA producing *B. subtilis* ΔxylAB and γ-PGA producing *B. subtilis* PG10 with different strength of xylose induction. The separation according to the metabolome analysis **(A)**, the observed versus predicted γ-PGA values **(B)**, the VIP values **(C)** and loading data **(D)** for metabolites highly connected to γ-PGA production rate in xylose induction experiments are shown for different cultivation conditions with varying γ-PGA production rates (*B. subtilis* Δxyl with 0 and 200 mM xylose as controls, *B. subtilis* PG10 with 0, 1, 5, 10, 15, 25, 40, 50, 80, 100, and 200 mM xylose). The intensity of the blue color increases with increasing γ-PGA production rate. ADP: adenosine 5′-diphosphate; AICAR: 5′-phosphoribosyl-5-amino- 4-imidazolecarboxamide, AMP: adenosine 5′-monophosphate, G6P: glucose-6-phosphate, IMP: inosine 5′-monophosphate, MEP: methyerythritol 4-phosphate, PRPP: 5-phospho-alpha-D-ribose 1-diphosphate, R1P: ribose-1-phosphate, R5P: ribose-5-phosphate, SBP: sedoheptulose 1,7-bisphosphate, UDP-Glc: uridine 5′-diphosphate-glucose, XMP: xanthosine 5′-phosphate.

For metabolome analysis, intracellular metabolites for 13 conditions (*B. subtilis ΔxylAB* with 0 and 200 mM xylose as controls, *B. subtilis* PG10 with 0, 1, 5, 10, 15, 25, 40, 50, 80, 100, and 200 mM xylose) were analyzed. For each strain and condition, the intracellular concentration of 77 metabolites was determined. The γ-PGA production was calculated based on the correlation between turbidity and xylose concentration obtained from Figure 4. Based on the obtained values for γ-PGA production under the investigated cultivation conditions, a PLS analysis was carried out (Figure 5A). The PLS analysis resulted in a model with R^2^ of 0.92 and Q^2^ of 0.87 (see Figure 5B). The samples were separated in t[1] according to the γ-PGA production rate.

Analogously to the promoter library, metabolites with varying concentrations for differing γ-PGA production rates could be identified (Figure 5). The metabolite concentrations with loading values higher than 0 increase with an increasing production rate. Comparing the two approaches for controlling the γ-PGA production rate, the promoter library and the xylose induction, the identified metabolites with high loading values partly differ. This fact is most likely caused by differences in growth rates and γ-PGA production rates for both approaches. Differences in metabolic demand (Koebmann et al., 2005) result in changes of metabolic fluxes and, hence, in metabolite concentrations. Changes in flux distribution have also been observed due to differing pH of the growth media and a negative correlation between acetoin and 2,3-butandiol and γ-PGA production was reported (Zhu et al., 2013). Regardless of the differences between the two approaches, several amino acids exhibit increased concentrations in both approaches. Especially phenylalanine increased with increasing promoter strength as well as increasing inducer concentration.

**FIGURE 6:**
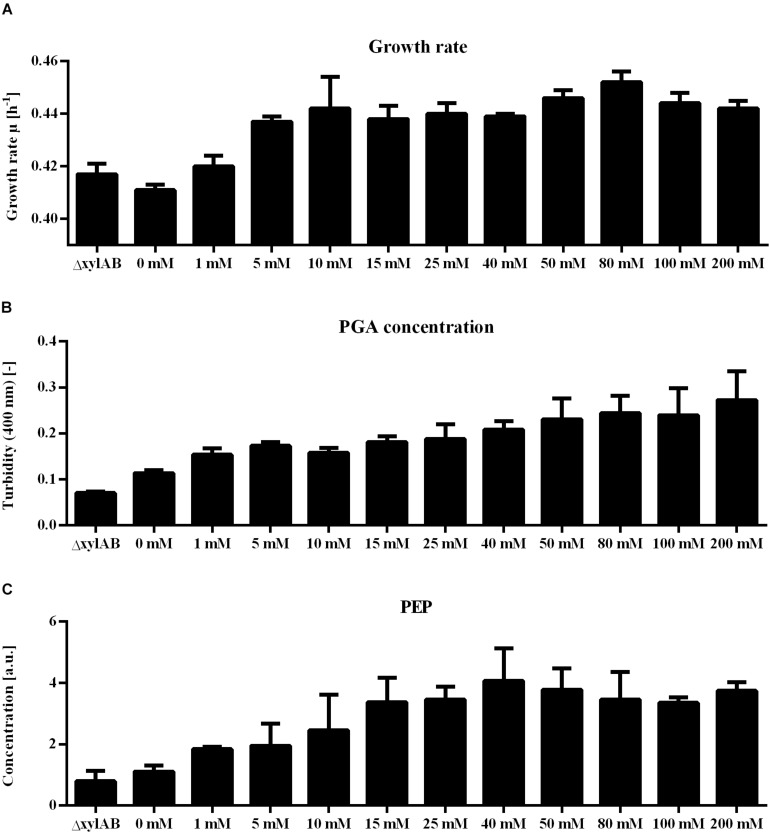
Phenotypic analysis of non-γ-PGA producing *B. subtilis* ΔxylAB and γ-PGA producing *B. subtilis* PG10 with different strength of xylose induction. The analysis of growth rates **(A)**, γ-PGA production **(B)** and intracellular phosphoenolpyruvate (PEP) concentration **(C)** are shown for different cultivation conditions with varying γ-PGA production rates (*B. subtilis* Δxyl with 0 mM xylose as control, *B. subtilis* PG10 with 0, 1, 5, 10, 15, 25, 40, 50, 80, 100, and 200 mM xylose).

**FIGURE 7:**
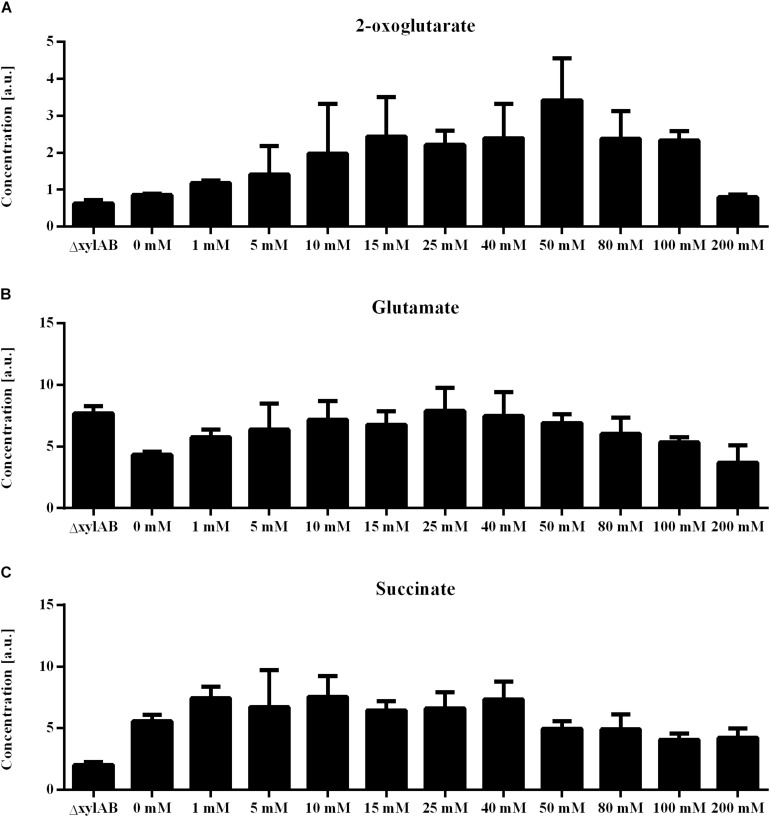
Metabolite concentrations for non-γ-PGA producing *B. subtilis* ΔxylAB and γ-PGA producing *B. subtilis* PG10 with different strength of xylose induction for metabolite extraction during the exponential growth phase. The intracellular concentrations of 2-oxoglutarate **(A)**, glutamate **(B)**, and succinate **(C)** are given for different cultivation conditions with varying γ-PGA production rates (*B. subtilis* Δxyl with 0, and 200 mM xylose as controls, *B. subtilis* PG10 with 0, 1, 5, 10, 15, 25, 40, 50, 80, 100, and 200 mM xylose).

Further, for the xylose induction approach mostly metabolites that accumulate for higher γ-PGA production rates were identified. In this approach, the increase in γ-PGA production rate is interconnected with a slight increase in growth rate (Figure 6). Here, phosphoenolpyruvate (PEP) is the metabolite exhibiting the highest correlation with γ-PGA production. The PEP concentration follows the increasing trend of both γ-PGA production rate and growth rate for increasing xylose induction strength. Besides PEP, further intermediates of the central carbon metabolism increase simultaneously with the γ-PGA production rate. Several metabolites originating from glycolysis, pentose phosphate pathway and TCA cycle like G6P, R5P and 2-oxoglutarate belong to the group of metabolites with high loading values. Hence, the increased metabolite concentrations are likely due to an increased overall carbon flux and not directly due to the γ-PGA production rate. However, the higher γ-PGA production rate with increasing xylose concentration increases the demand for a higher flux through the metabolism resulting in higher glucose uptake rates as indicated by the increasing G6P concentrations. Among the metabolites that are negatively related to the higher γ-PGA production rate, glutamine is the most striking one. Since glutamine is a substrate for *de novo* synthesis of glutamate from glucose, its concentration is strongly connected to the demand for glutamate. The increasing demand for glutamate with higher γ-PGA production also influences the proline and NADP concentrations as indicated by the loading values of these metabolites. Succinate is a further metabolite indicating the changes in glutamate demand since the carbon flux at the 2-oxoglutarate branch point can either be directed toward glutamate or succinate. The obtained positive loading value for succinate indicates an increase of the concentration with increasing xylose induction. However, the absolute loading value is low emphasizing a weaker connection of the succinate concentration to the γ-PGA production rate. The comparison of concentrations for 2-oxoglutarate, succinate and glutamate (Figure 7) demonstrates the connection. First, the effect of higher overall carbon fluxes can also be seen for these metabolite concentrations. The concentrations of these metabolites increase to a certain extent with higher xylose concentration. While 2-oxoglutarate is increasing for xylose concentrations up to 50 mM, the glutamate and succinate concentrations are already decreasing from 25 and 10 mM on. Therefore, the positive loading value for succinate is mostly reasoned in the small concentration for the reference and increasing concentration for low xylose concentrations up to 10 mM. Additionally, the concentrations of the three metabolites at the 2-oxoglutarate branch point reflect the increasing carbon flux to glutamate for stronger induction. This effect is emphasized by the fact that succinate starts decreasing for weaker induction than glutamate. The metabolite measurement results match the increasing growth rate and γ-PGA production rate for the xylose induction approach suggesting higher metabolic fluxes caused by the demand for glutamate for γ-PGA production.

## Discussion

Poly-γ-glutamic acid production with glucose as sole carbon source requires strong flux rerouting in the metabolic network of *B. subtilis*. We studied the metabolic consequences of γ-PGA production using metabolome analysis of the newly generated strains. Several studies reported an increase of γ-PGA titer by the addition of exogenous glutamate (Lin et al., 2016), suggesting *de novo* glutamate synthesis as bottleneck for γ-PGA production. Yao et al. (2010) investigated the origin of carbon in γ-PGA using 13C-labeled glucose and L-glutamate as carbon sources for a *B. subtilis* producer. Maximal 9% of the γ-PGA was derived from glucose. For the efficient use of glucose as sole carbon source, the metabolic bottlenecks limiting γ-PGA production need to be determined. Here, *B. subtilis* strains with varying γ-PGA production rates were subjected to metabolome analysis to identify possible bottlenecks.

The strength of γ-PGA synthetase expression was varied by two approaches: a library of constitutive promoters with differing expression strength and a xylose-inducible promoter that allowed titrating of expression strength. Notably, as the growth rate and PGA synthesis rate of *B. subtilis* differed between the different approaches, the metabolome analyses differed. Several metabolites like R5P were positively correlated during xylose induction while negatively correlated with increasing constitutive *pgs* expression using the promoter library. Especially the concentration of R5P was previously shown to increase with increasing growth rate as the RNA synthesis rate correlates positively with the growth rate. In eukaryotic cells, the intracellular R5P concentration was reported to influence the rate of *de novo* purine synthesis (Pilz et al., 1984). A higher synthesis rate of purine is interconnected with a higher growth rate since the RNA synthesis rate increases with the growth rate. Even the rate of glycolysis is partially controlled by the R5P concentration via the activity of pyruvate kinase in *B. licheniformis* (Tanaka et al., 1995) and *E. coli* (Waygood et al., 1975). The enzyme is activated by high R5P concentrations resulting in a higher flux through glycolysis when R5P accumulates (Sakai et al., 1986). Influencing the flux through glycolysis and the growth rate, the increasing R5P concentrations observed for higher inducer concentrations is in line with the suggestion of increasing overall fluxes. Notably, the growth rate is concurrently increasing with the γ-PGA production rate (Figure 6). The increasing demand for glutamate results in a higher total carbon flux. A higher growth rate is mostly connected with a higher glucose uptake rate as reflected by the increasing G6P concentrations for higher xylose induction. This positive correlation between metabolite concentration and γ-PGA production rate was not unique for R5P. These results suggest that metabolism is reacting on the increased demand for glutamate by increasing overall flux. However, besides the γ-PGA production rate the growth rate is also increasing. Based on these results, a higher γ-PGA production using this chassis might be achieved by limiting the growth rate, while maintaining or even further increasing the rate of γ-PGA production.

For the promoter library, the γ-PGA titers were lower compared to the values obtained from the xylose inducible promoter. Also, with increasing expression and hence increasing PGA production the growth rate did not differ. For the constitutive promoters, the changes in metabolite concentrations can be solely attributed to the change in γ-PGA production. Besides the already discussed R5P further metabolites like serine and DHAP exhibited negative loading values, indicating a negative correlation of metabolite concentration and PGA production. Like R5P, serine is also known to allosterically control the pyruvate kinase activity (Amelio et al., 2014). The decreasing concentrations of both serine and R5P might therefore lead to a decreasing flux through glycolysis. However, acetyl-CoA is accumulating at higher γ-PGA production rates (Figure 3C). Acetyl-CoA is a substrate of the citrate synthase encoded by *citZ* (Fletchner and Hanson, 1969). The accumulation of acetyl-CoA suggests that the activity of the citrate synthase is limiting glutamate synthesis. Regardless of the γ-PGA production rate, the glutamate concentration was detected to be constant. In *B. subtilis*, the glutamate concentration is controlled by six regulator proteins (Sonenshein, 2007). As glutamate is a key metabolite in the carbon and nitrogen metabolism of the cell, the homeostatic glutamate concentration is beneficial for the cell. But the higher demand for glutamate is reflected in changes of metabolite concentrations that are not necessarily precursors for glutamate synthesis.

The metabolome analysis revealed that many nucleotides and intermediates of the purine metabolism are inversely related to the γ-PGA synthesis rate. In bacteria, nucleotides are known to participate in cell signaling and regulation as second messengers (Kalia et al., 2013). Moreover, the mentioned nucleotides are precursors for the synthesis of secondary metabolites. *B. subtilis* is especially known for industrial production of riboflavin (Perkins et al., 1999). The decreasing concentrations of intermediates from the purine pathway might therefore also be caused by the higher demand for carbon flux toward glutamate instead of other cellular products. Further direction of the carbon flux for γ-PGA synthesis might be achieved by limiting the ability of the strain to produce metabolites derived from purine metabolism.

The amino acids arginine, glutamine and proline are derived from glutamate, and therefore their synthesis is competing with γ-PGA production. The constant glutamate level during higher glutamate consumption resulted in decreased concentrations of the amino acids derived from glutamate. While arginine decreases for the promoter library approach, proline and glutamine have been determined as metabolites with negative loading values for the inducible promoter approach. But these three amino acids are involved in different regulatory systems. The global regulator of nitrogen metabolism in *B. subtilis*, TnrA, senses nitrogen availability based on the level of glutamine. TnrA forms a complex with the glutamine synthetase (Wray et al., 1996, 2001). It represses the glutamate synthase and glutamine synthetase genes. Hence, the decreasing glutamine concentration enables a higher glutamate synthase activity. During the conversion of arginine to glutamate, ornithine is formed as an intermediate. High ornithine concentrations activate the RocR protein (Belitsky and Sonenshein, 1999). In the *B. subtilis* genome the glutamate dehydrogenase *rocG* is located upstream of the *rocABC* operon which is involved in arginine-ornithine-proline metabolism (Gardan et al., 1997). The *rocG* gene is co-regulated with the *rocABC* operon by binding of the RocR regulator. Hence, RocR increases the glutamate dehydrogenase expression. This enzyme converts glutamate to 2-oxoglutarate. For higher γ-PGA production with the inducible promoter, arginine and proline exhibit reduced concentrations. These decreasing concentrations most likely also result in decreased ornithine concentrations since ornithine is an intermediate in arginine and proline synthesis. As a result of decreased ornithine concentrations, the glutamate dehydrogenase activity decreases. The decreased glutamate dehydrogenase activity limits the decrease in glutamate concentration even for higher γ-PGA production rates.

Glutamate *de novo* synthesis requires NADPH as a cofactor of glutamate synthase. The main NADPH supplying reactions are found in the pentose phosphate pathway (Zamboni et al., 2004). Metabolite analysis revealed higher concentrations for NAD(P)^+^ with increasing γ-PGA production rate for strains with the constitutive promoter. While the analysis method does not detect the actual redox status in the cell, the increasing total concentration of NAD(P)^+^/NAD(P)H points out the enhanced demand for redox equivalents for the production of γ-PGA.

Succinyl-CoA exhibits a highly positive loading value for the promoter library approach. In the TCA cycle, 2-oxoglutarate is converted to succinyl-CoA by the 2-oxoglutarate dehydrogenase. The increasing glutamate consumption requires a higher flux from glucose to 2-oxoglutarate. Since the branch point of 2-oxoglutarate and its conversion to glutamate is tightly regulated, the increasing succinyl-CoA concentration is likely caused by increasing fluxes to 2-oxoglutarate that are not completely directed to glutamate but also to succinyl-CoA.

High titer γ-PGA production mostly involves a mixture of carbon sources like glycerol, citrate, and glutamate as provided in medium E commonly used for γ-PGA production (Leonard et al., 1958). γ-PGA production cost can be reduced by utilization of glucose as sole carbon source. However, in *B. licheniformis* metabolome analysis revealed the limitation of γ-PGA synthetase expression when glucose is used as carbon source instead of glycerol (Mitsunaga et al., 2016). In this study, the γ-PGA synthesis was independent from the native expression regulation by integration of a constitutive and inducible promoter. Aiming at an inexpensive γ-PGA production, the provided information will be used to guide metabolic engineering for improved γ-PGA production from glucose as sole carbon source in further studies.

## Data Availability Statement

All datasets generated for this study are included in the article/Supplementary Material.

## Author Contributions

BH and LB conceived and designed the study. SP and EF contributed to the design and data analysis of metabolomics experiments. BH performed the experiments and drafted the manuscript. LB, SP, and EF revised the manuscript and approved the final manuscript.

## Conflict of Interest

The authors declare that the research was conducted in the absence of any commercial or financial relationships that could be construed as a potential conflict of interest.
