# Supplementary material for: Identification of Key Metabolites in Poly-γ-Glutamic Acid Production by Tuning γ-PGA Synthetase Expression
Source: Front Bioeng Biotechnol. 2020 Jan 30;8:38. doi: 10.3389/fbioe.2020.00038 (PMC7002566; doi:10.3389/fbioe.2020.00038)
Supplement: Supplementary file 1 [file Table_1.DOCX]

Supplementary Material

# Supplementary Table

Supplementary Table 1: Primers used in this study

| **name** | **Sequence (5’-3’)** | **Purpose** |
| --- | --- | --- |
| BS-25 | GGGCGTGTCAATAATATCACTC | Vector backbone linearization |
| BS-26 | GGGCGACGGATGGTGATCCC | Vector backbone linearization |
| BS-21 | GGGATCACCATCCGTCGCCCGAAGCGGATAACGTACTTC | Amplification of TS1 for pBs-08 |
| BS-22 | TAAAGCACCCGTTACTAAGCGAAGGAAGC | Amplification of TS1 for pBs-08 |
| BS-23 | GCTTAGTAACGGGTGCTTTACGGCCTAAAT | Amplification of TS2 for pBs-08 |
| BS-24 | GTGATATTATTGACACGCCCTGTCAGGCATTCGTTATCG | Amplification of TS2 for pBs-08 |
| TS1_fwd | GGGATCACCATCCGTCGCCCGTCCGATCAGTCGGGATTC | Amplification of TS1 for pBs-01 |
| TS1_rev | TCTTGTCAGCCTGATATCCTTCTGGACGTATAG | Amplification of TS1 for pBs-01 |
| P(veg)_fwd | AGGATATCAGGCTGACAAGAGAGAAAGG | Amplification of Pveg for pBs-01 |
| P(veg)_rev | CACATTGCTTTCCACCTCACTACATTTATTG | Amplification of Pveg for pBs-01 |
| TS2_fwd | GTGAGGTGGAAAGCAATGTGGTTACTCATTATAGC | Amplification of TS2 for pBs-01 |
| TS2_rev | GTGATATTATTGACACGCCCGGCGCATTCAGCATTCCC | Amplification of TS2 for pBs-01 |
| BS-09 | CCAGAAGGATATCAGGCTGACAAGACAGATAATTTTGCTCTTGAAATAG | Amplification of Pxyl for pBs-02 |
| BS-10 | CACATTGCTTTCCACCTCACTTCATATAGTAAGTACATCACCTATTAG | Amplification of Pxyl for pBs-02 |
| BS-47 | GAAGCCTCAGCGTCCAAG | Verification of pBs-08 deletions |
| BS-48 | ATCAGCAGCTCCGGATCG | Verification of pBs-08 deletions |
| BS-57 | GCGTTCGCGTTTCTTCGTG | Verification of Pveg integration |
| BS-58 | CCGCATAGCTCTCCTATGCAG | Verification of Pveg integration |
|  |  |  |
| BS-109 | GGGATCACCATCCGTCGCCCGCTTGGAGGTATTGAAAC | Amplification of P(veg) for promoter library |
| BS-110 | TTACGCATGCGGCTCCACCTCACTACATTTATTG | Amplification of P(veg) for promoter library |
| BS-113 | ACTGAATTATGAGAACTCTCGAACCCGAATG | Amplification of *amyE* for pBs-21 |
| BS-114 | ACAGAGTGATATTATTGACACGCCCAAGTCCCGTCTAGCCTTG | Amplification of *amyE* for pBs-21 |

| BS-115 | GGAAGCGGAAGAATGAAG | Amplification of Spc^R^ for pBs-21 |
| --- | --- | --- |
| BS-116 | TATATCAACGGGCACTGGCCGTCGTTTTAAC | Amplification of Spc^R^ for pBs-21 |
| BS-117b | GGCCAGTGCCCGTTGATATAATTTAAATTTTATNNGACAAAAATGG | Amplification of promoter library |
| BS-118 | AAGTCCCGTCTAGCCTTG | Amplification of promoter library |

| BS-180 | AATTTTATAAGACAAAAATGGGCTCGTG | Amplification of promoter PV35.1 |
| --- | --- | --- |
|  |  |  |
| BS-182 | AATTTTATGTGACAAAAATGGGCTCGTG | Amplification of promoter PV35.26 |
| BS-193 | AATTTTATTTTACAAAAATGGGCTCGTG | Amplification of promoter PV35.3 |
